# Appsolutely secure? Psychometric properties of the German version of an app information privacy concerns measure during COVID-19

**DOI:** 10.3389/fpsyg.2022.899092

**Published:** 2022-07-22

**Authors:** Samuel Tomczyk

**Affiliations:** Department of Health and Prevention, Institute of Psychology, University of Greifswald, Greifswald, Germany

**Keywords:** privacy concerns, mHealth, assessment, COVID-19, validation, apps, cross-sectional, contact tracing

## Abstract

**Introduction:**

Privacy concerns are an important barrier to adoption and continued use of digital technologies, particularly in the health sector. With the introduction of mobile health applications (mHealth apps), the construct of app information privacy concerns has received increased attention. However, few validated measures exist to capture said concerns in population samples, although they can help to improve public health efforts.

**Methods:**

Using a cross-sectional survey of German adults (mean age = 35.62; 63.5% female), this study examined psychometric properties of the app information privacy concerns scale (AIPC). Analyses comprised confirmatory factor analysis, factorial validity (exploratory factor analysis), internal consistency, convergent validity (i.e., correlations with privacy victimhood, and app privacy concerns), and discriminant validity (i.e., daily app use, adoption intentions, and attitudes toward COVID-19 contact tracing app use).

**Results:**

The analysis did not support the proposed three-factor structure of the AIPC (i.e., anxiety, personal attitude, and requirements). Instead, a four-factor model was preferable that differentiated requirements regarding disclosure policies, and personal control. In addition, factors mirroring anxiety and personal attitude were extracted, but shared a significant overlap. However, these factors showed good reliability, convergent and discriminant validity.

**Discussion:**

The findings underline the role of app information privacy concerns as a significant barrier to mHealth app use. In this context, anxiety and personal attitudes seemed particularly relevant, which has implications for health communication. Moreover, the observed differentiation of external (disclosure) and internal (control) requirements aligns with health behavior change models and thus is a promising area for future research.

## Introduction

Today, ubiquitous computing is a reality, and smart mobile devices enable us to work, communicate and interact everywhere (Abowd and Mynatt, 2000; Friedewald and Raabe, 2011). Mobile applications or apps as technological interfaces for end-users fulfill different functions, and offer a variety of possibilities: organizing apps (e.g., calendars, to-do-lists), informational and communication apps (e.g., news channels, WhatsApp), entertainment apps (e.g., mobile games), and mixed apps (e.g., educational games, social media apps with organizing functions) are some examples (e.g., Hew et al., 2015). Their popularity and reach have led to increased interest in mobile health (*mHealt*h) in recent years, with apps addressing prevention (e.g., supporting a healthy diet), treatment (e.g., monitoring medication adherence), and recovery (e.g., providing tips for physical activity following surgery). MHealth apps comprise self-administered apps but also monitoring apps, and diagnostic tools to support medical decisions, and the translation of treatment effects into everyday life (e.g., Martínez-Pérez et al., 2014; Byambasuren et al., 2018; Chib and Lin, 2018; Hensher et al., 2021; Grundy, 2022).

In Germany, since late 2019, new legislation (so-called *Digitale Versorgung Gesetz*) allows medical professionals to prescribe mHealth apps as medical devices (e.g., for depressive disorders, or to monitor blood pressure). While this approach to public health and personalized medicine is commendable (Grundy, 2022), it also leads to questions about data security, privacy, and commercialization of health (Martínez-Pérez et al., 2014). In fact, many apps gather (sensitive) personal data, and the more data, the more precise the customization to individual needs, which supports successful treatment processes. Hence, highly customizable apps require a trade-off between information privacy and comfort or customization (Jeminiwa et al., 2019; Iwaya et al., 2020; Hensher et al., 2021; Grundy, 2022). Information privacy refers to the degree of autonomous and self-directed disclosure of private information (Smith et al., 2011). App-based data therefore has a high economic value and needs far-reaching protection. From a user-centered design perspective, establishing transparent privacy policies and data security measures is paramount (Azhar and Dhillon, 2016; Adjekum et al., 2018; Jeminiwa et al., 2019). Moreover, from an end-user perspective, it is important to trust data security protocols, and a lack of trust can impede uptake and continued use of digital health technologies, such as apps (Schnall et al., 2015; Azhar and Dhillon, 2016). Frameworks like the unified theory of acceptance and use of technology (UTAUT; e.g., Venkatesh and Davis, 2000; Venkatesh et al., 2003, 2012) have adopted issues like privacy concerns and lack of trust in technologies as perceived barriers of use. Consequently, many studies using the UTAUT framework have also examined these constructs (Williams et al., 2015; Dwivedi et al., 2020).

### Literature review of information privacy measures in mobile applications

Although the literature on information privacy and data security provides a variety of measures and constructs to measure end-user attitudes, few instruments focus on apps (Bélanger and Crossler, 2011; Li, 2011; Dinev et al., 2015; Benjumea et al., 2020). So far, many studies have analyzed privacy policies of app providers, reviewed or suggested measures or content of data security statements and policies. However, real-world evaluations of these suggestions are scarce (e.g., Li, 2011; Sunyaev et al., 2015; Chib and Lin, 2018; Benjumea et al., 2020; Iwaya et al., 2020; Grundy, 2022). Consequently, studies should focus on user perceptions of information privacy. Previous research has produced instruments measuring general concern for information privacy in the population, in internet users, and in mobile users (Bélanger and Crossler, 2011; Li, 2011). These measures usually capture attitudes toward data collection, storage, and surveillance, perceived personal control, and (fear of) secondary use of information. So far, only one measure, the app information privacy concern scale (AIPC) includes all of these aspects regarding mobile applications (Buck and Burster, 2017). It synthesizes previous work on Concerns for Information Privacy (Smith et al., 1996), Internet Users’ Information Privacy Concerns (Malhotra et al., 2004), and Mobile Users’ Information Privacy Concerns (Xu et al., 2012). Buck and Burster (2017) developed the measure in a three-step process: They started with items from Internet Users’ Information Privacy Concerns measure (Malhotra et al., 2004), which was based on Concerns for Information Privacy (Smith et al., 1996). The measure describes collection (i.e., concern about an imbalance of costs and benefits regarding data sharing *via* services), control (i.e., perceived control over personal information and data use), and awareness (i.e., awareness about organizational information privacy practices). Then, they extended the model by including Mobile Users’ Information Privacy Concerns (Xu et al., 2012), specifically, concerns about perceived surveillance, intrusion, and secondary use of information. In a third step, they added an item measuring general information privacy concerns (I am concerned about threats to my personal privacy today) based on Smith et al. (1996). Their analysis of the instrument resulted in a three-factor model with the factors anxiety (factor 1), personal attitude (factor 2), and requirements (factor 3). Anxiety describes concerns regarding collection, secondary use of data, and surveillance. Personal attitude refers to preferences regarding information and disclosure, and requirements refer to request toward third parties about data handling.

To date, though, the scale has not been validated in other samples and applied contexts. Hence, this study presents a validation of the German version of the AIPC in a community sample. As context, this study addresses the use of a Corona virus tracing app (e.g., the Corona Warn-App) as a use case of app information privacy. Contact tracing apps are technological solutions that support infection prevention and public health efforts, and more than 100 countries use(d) contact tracing apps during the COVID-19 pandemic (Gupta et al., 2021). Contact tracing apps require users to agree to surveillance and contact tracing *via* their smart device. The apps inform users about contact with positive (infected) cases, suggest adequate preventive and mitigation measures, and allow governmental institutions to define containment or hotspot zones (Kahnbach et al., 2021). Previous studies examined barriers and facilitators of adopting tracing app use and showed that they are effective in reducing infection rates (e.g., Jenniskens et al., 2021; Kahnbach et al., 2021; Kolasa et al., 2021). However, tracing apps often do not provide sufficient information about personal data breaches, which might increase privacy concerns and subsequently, reduce use intentions (Jenniskens et al., 2021). Hence, contact tracing apps are an important use case to investigate privacy concerns regarding mobile health apps. Therefore, this study examines psychometric properties of the German version of the AIPC in the context of the COVID-19 pandemic.

## Materials and methods

Between May and July 2020, data was collected *via* an online survey on adopting a COVID-19 tracing app. Recruitment efforts comprised social media posts (Facebook groups, corona-related websites, YouTube), press outlets (local news report, Press, and Media Relations Office of the University), and personal communications. The survey was pretested *via* cognitive debriefings of a small sample (*n* = 20) to ensure clarity, readability, accessibility, and proper functioning. During the pretest, participations took between 10 and 60 min to complete the survey (depending on literacy, familiarity with surveys, etc.). Therefore, a time frame of 10–60 min was defined as a rule of thumb to identify outliers. The survey captured a period of about 4 weeks before and after the launch of the governmentally supported COVID-19 tracing app, the Corona Warn-App (June 16). The survey comprised questions about adoption intentions, motivations and barriers of tracing app use, including information privacy concerns. While a previous study (Tomczyk et al., 2021) focused on predictors of and barriers to tracing app use (with privacy concerns as one of many variables), this study inspects psychometric properties of the AIPC scale to assess its utility for the field. As a frame of reference for contact tracing apps, we described the functionality of contact tracing apps as (i) monitoring and tracking infection chains, (ii) delivering immediate support and information in case of an infection or contact with an infected person, and (iii) and possibly, providing support for persons in quarantine by monitoring health, and tailoring information and preventive actions.

### Sample

In sum, 593 persons took part in the survey. After excluding speeders, that is participants who completed the survey in less than 10 min or showed monotone response patterns for >80% of the questions, 349 participants remained (mean age = 35.62, SD = 14.66; range = 18–82 years; 63.5% female). On average, participants completed the survey in 22.65 (SD = 7.93) minutes. Participants could enter a raffle to win one of fifty gift vouchers (€15 each) as an incentive. Having completed the study, participants received additional information on COVID-19 and tracing apps, including several hyperlinks to freely available tracing apps. The local Ethics Committee approved the study procedure. Items of the survey are accessible as Supplementary Material of a previous publication (Tomczyk et al., 2021).

### Measurement instruments

#### Sociodemographic data

Sociodemographic data comprised age, gender [1 (female), 2 (male)], number of persons in one’s household, current level of education [1 (upper secondary education, i.e., “Abitur” or higher educational achievement), 0 (lower secondary education or less)], region [0 (rural, i.e., up to 10,000 inhabitants), 1 (urban, i.e., up to 100,000 inhabitants), 2 (metropolitan, i.e., over 100,000 inhabitants); dummy-coded with rural as a reference category)], and migration background [1 (father/mother/participant born in Germany), 2 (father/mother/participant born elsewhere)].

#### App information privacy concerns

The AIPC scale (German version of the scale provided *via* personal communication; Buck and Burster, 2017) comprises seventeen items (α = 0.91), for instance, “A good privacy policy for mobile app users should have a clear and conspicuous disclosure” (see Table 1). The response scale is a seven-point Likert scale. For the analysis, we performed a confirmatory factor analysis of the three factors suggested by Buck and Burster (2017). However, we also tested factorial validity of the AIPC *via* an exploratory factor analysis. We used mean values of relevant subscales for statistical comparisons. Although it is recommended to perform exploratory and confirmatory factor analysis in different samples (Hurley et al., 1997), this study aims to examine psychometric properties of the original scale developed by Buck and Burster (2017). Given the differences in sample composition, both analyses are included to illustrate the impact of these differences and inform future research.

**TABLE 1:** Items of the app information privacy concerns scale.

Item	Text
1	I am concerned that mobile apps are collecting too much information about me.
2	I believe that as a result of my using mobile apps, information about me that I consider private is now more readily available to others than I would want.
3	I am concerned that mobile apps may monitor my activities on my mobile device.
4	I feel that as a result of my using mobile apps, information about me is out there that, if used, will invade my privacy.
5	I am concerned that mobile apps may use my personal information for other purposes without notifying me or getting my authorization.
6	I am concerned that mobile apps may share my personal information with other entities without getting my authorization.
7	When I give personal information to use mobile apps, I am concerned that apps may use my information for other purposes.
8	I am concerned about threats to my personal privacy today.
9	It is very important to me that I am aware and knowledgeable about how my personal information will be used.
10	When mobile apps ask me for personal information, I sometimes think twice before providing it.
11	To me, it is the most important thing to keep my privacy intact from app providers.
12	Compared to others, I am more sensitive about the way mobile app providers handle my personal information.
13	A good privacy policy for mobile app users should have a clear and conspicuous disclosure.
14	Mobile app providers seeking information online should disclose the way the data are collected, processed, and used.
15	(Mobile app user) control of personal information lies at the heart of mobile app users’ privacy.
16	Mobile app privacy is really a matter of consumers’ right to exercise control and autonomy over decisions about how their information is collected, used, and shared.
17	It usually bothers me when mobile apps ask me for personal information.

#### Convergent validity measures

To test convergent validity, direct and indirect experiences of privacy victimhood and data misuse were measured on a five-item scale (α = 0.91; e.g., How frequently have you personally been the victim of what you felt was an improper invasion of privacy?). Items were rated on a five-point Likert scale from 0 (never) to 5 (very frequently) and based on previous research on information privacy concerns (Xu et al., 2012). Furthermore, an open-ended question asked participants why they might not use a contact tracing app. Responses were coded to reflect tracing app privacy concerns [1 (yes), 0 (no)] as a reason for non-use.

#### Discriminant validity measures

Discriminant validity measures included daily app use, adoption intentions, and attitudes toward COVID-19 tracing apps. Intentions comprised a scale of three items [e.g., I plan to use a tracing app within the next 3 months; 1 (highly unlikely) to 7 (highly likely); (α = 0.99)]. Attitudes were measured *via* a four-item scale (e.g., good-bad, helpful–not helpful) on a 7-point semantic differential, recoded to represent positive attitudes (α = 0.89). An open-ended question captured daily smartphone app use (in hours).

### Statistical analysis

First, descriptive statistics of sociodemographic and attitudinal data were inspected. Second, a confirmatory factor analysis tested the three-factor model proposed by Buck and Burster (2017). Model fit indices (Chi Square test, CFI, TLI, and RMSEA; Schreiber et al., 2006) are reported. A non-significant Chi Square test (*p* > 0.05), CFI greater than 0.95, TLI greater than 0.90, and RMSEA lower than 0.08 indicate good model fit (Schreiber et al., 2006). Third, an exploratory factor analysis of the AIPC using varimax rotation, with a KMO > 0.70 as quality indicator (Dziuban and Shirkey, 1974) was performed to test factorial validity. Fourth, reliability was tested *via* internal consistency (Cronbach’s α) for the AIPC and the subscales. Fifth, for convergent and discriminant validity, correlations of AIPC values with experiences of privacy victimhood and app privacy concerns (convergent validity) as well as daily app use, intentions, and attitudes toward tracing app use (discriminant validity) were examined. Descriptive statistics and correlations were calculated with SPSS version 27 (RRID: SCR_016479), factor analyses with Mplus version 8 (Muthén and Muthén, 1998-2017, RRID: SCR_015578). All analyses assumed α = 0.05.

## Results

### Descriptive statistics

The sample consisted of 349 participants (*M*_*age*_ = 35.62 years; 65.3% female) with mostly higher secondary education, from urban or metropolitan regions, and without a migration background (77.4%). Overall, app information privacy concerns were rather high, yet a minority (*n* = 30; 8.6%) explicitly stated privacy concerns as a main reason for non-use of the contact tracing app (see Table 2).

**TABLE 2:** Descriptive statistics of sociodemographic data and attitudinal variables in the analysis sample (*N* = 349).

	Total (*N* = 349) [*n* (%) or mean (SD)]
**Sociodemographic data**	
Age (range: 18–82)	35.62 (14.66)
Gender (female)	226 (65.30)
Persons per household	2.53 (1.58)
**Education**	
≤Lower secondary	55 (16.50)
Upper secondary	278 (83.50)
**Region**	
Rural	66 (20.10)
Urban	143 (43.60)
Metropolitan	119 (36.30)
Migration background^a^	79 (22.60)
**Attitudinal variables**	
**App information privacy concerns (range: 1–7)**	
Anxiety	5.55 (1.12)
Personal attitudes	5.52 (1.12)
Requirements	6.12 (0.70)
Privacy victimhood (range: 1–5)	2.33 (0.83)
Privacy concerns (yes, as a barrier to tracing app use)	30 (8.60)
Adoption intentions of tracing app use (range: 1–7)	3.66 (2.37)
Attitudes toward tracing app use (range: 1–7)	4.19 (1.65)
Daily smartphone app use (hours per day)	2.63 (1.78)

^a^Either the respondent, their mother or their father were not born in Germany.

### Confirmatory factor analysis

The confirmatory factor analysis showed a poor fit of the three-factor model [χ^2^ = 722.66, *df* = 116, *p* = < 0.001; CFI = 0.84, TLI = 0.81, and RMSEA = 0.12, 90% CI (0.11,0.13)]. In this model (see Supplementary Table 1), standardized factor loadings were acceptable [(i.e., above 0.5) for factor 1 (anxiety), and factor 2 (personal attitudes)]. However, factor loadings were low for items 15 (β = 0.245), 16 (β = 0.274), and 17 (β = 0.350), which were part of factor 3 (requirements). Interestingly, item 17 (It usually bothers me when mobile apps ask me for personal information) also showed poor fit in the original analysis by Buck and Burster (2017).

### Exploratory factor analysis

The exploratory factor analysis with varimax rotation showed a good fit of the data (KMO = 0.90), and explained about 59.8% of cumulative variance. However, not three but four factors reached an eigenvalue > 1 (see Table 3), which was confirmed by parallel analysis (with 10 replications). Except for one item, all items had a loading of >0.53 on at least one factor. Factor 3 (disclosure; 5.2% explained variance) and 4 (control; 4.6%) were distinct, but factor 1 (information; 41.7%) and 2 (data misuse; 8.3%) shared variance in items referring to the collection of personal data (item 1) as well as the concern about the misuse of information (items 5 to 7). According to the analysis, factor 1 comprised eight items, factor 2 five items, factor 3 and 4 two items each.

**TABLE 3:** Results of the exploratory factor analysis with varimax rotation of the app information privacy concerns scale (*N* = 349).

	Factor 1 (“information”)	Factor 2 (“data misuse”)	Factor 3 (“disclosure”)	Factor 4 (“control”)
*Item 1*	*0.608*	**0.626**	0.181	−0.055
Item 2	0.160	**0.687**	0.102	0.204
*Item 3*	**0.646**	*0.487*	0.140	0.041
Item 4	0.145	**0.710**	0.081	0.140
*Item 5*	*0.548*	**0.591**	0.318	−0.158
*Item 6*	*0.533*	**0.543**	0.337	−0.139
*Item 7*	**0.593**	*0.591*	0.264	−0.146
Item 8	**0.539**	0.341	0.078	−0.048
Item 9	**0.587**	0.148	0.402	0.160
Item 10	**0.582**	0.108	0.102	0.071
Item 11	**0.825**	0.111	0.181	0.076
Item 12	**0.716**	0.181	0.087	0.091
Item 13	0.184	0.113	**0.793**	0.122
Item 14	0.240	0.242	**0.719**	0.194
Item 15	0.063	0.099	0.066	**0.735**
Item 16	0.019	0.016	0.149	**0.719**
Item 17	**0.369**	0.271	0.156	−0.006
Explained variance	41.747	8.313	5.229	4.559
Eigenvalue (sample correlation matrix)	7.448	1.824	1.314	1.144
Eigenvalue (parallel analysis)	1.405	1.321	1.221	1.205

Highest factor loadings per item are printed **in bold type**; variables with high factor loadings on two separate factors are printed *in italic type.*

Compared to the original analysis by Buck and Burster (2017), factor 2 overlapped with the factor labeled *anxiety*, while factor 1 included all items of the factor named *personal attitude*, but also shared variance with items from factor 2. The original factor titled *requirements* was split in two: requirements regarding disclosure (factor 3) and control (factor 4).

### Internal consistency

The three-factor model showed very good (factor 1/anxiety: α = 0.91), good (factor 2/personal attitude: α = 0.82), and poor internal consistency (factor 3/requirements: α = 0.59). The four-factor model showed acceptable, (factor 4/control: α = 0.76; factor 2/data misuse: α = 0.79), good (factor 3/disclosure: α = 0.81), and very good internal consistency (factor 1/information: α = 0.92).

### Convergent and discriminant validity

Results of convergent and discriminant validity of the three-factor model and the four-factor model of app information privacy concerns are presented in Table 4. AIPC scores of the three-factor model correlated positively with privacy victimhood [*r*(347) = 0.36, *p* < 0.001] and negatively with daily app use [*r*(347) = −0.13, *p* = 0.020], adoption intentions [*r*(347) = −0.30, *p* < 0.001], and attitudes [*r*(347) = −0.36, *p* < 0.001]. The correlation with privacy concerns was not significant [*r*(347) = −0.01, *p* = 0.836]. In fact, privacy concerns correlated negatively only with attitudes [*r*(347) = −0.11, *p* = 0.046], the remaining associations were not significant. Concerning the four-factor model, factor 1 (information) and factor 2 (data misuse) showed similar convergent and discriminant validity compared to the three-factor model, but factor 3 (disclosure) and factor 4 (control) did not significantly correlate with any other variable.

**TABLE 4:** Bivariate correlations between app information privacy concerns, daily app use, privacy concerns as a barrier to tracing app use, privacy victimhood, adoption intentions, and attitudes toward COVID-19 contact tracing apps (*N* = 349).

	1	2	3	4	5	6	7	8	9	10	11	12
1. Anxiety	1											
2. Personal attitudes	0.68^c^	1										
3. Requirements	0.40^c^	0.44^c^	1									
4. Information	0.66^c^	0.93^c^	0.28^c^	1								
5. Data misuse	0.80^c^	0.19^c^	0.25^c^	0.14^b^	1							
6. Disclosure	0.27^c^	0.26^c^	0.55^c^	0.08	0.06	1						
7. Control	−0.04	14^b^	0.73^c^	−0.01	−0.02	0.06	1					
8. Privacy victimhood	0.40^c^	0.32^c^	0.06	0.33^c^	0.28^c^	−0.03	−0.04	1				
9. Privacy concerns	−0.04	0.03	0.03	0.01	−0.06	0.03	0.05	0.03	1			
10. Daily app use (hours)	−0.11	−0.20^c^	−0.01	−0.22^c^	0.01	0.03	0.03	−0.09	0.05	1		
11. Adoption intentions	−0.33^c^	−0.26^c^	−0.05	−0.29^c^	−0.24^c^	0.03	0.10	−0.18^ c^	−0.07	0.10	1	
12. Attitudes	−0.35^c^	−0.38^c^	−0.11^a^	−0.39^c^	−0.18^c^	−0.02	0.06	−0.24^ c^	−0.11^a^	0.06	0.66^c^	1

^a^*p* < 0.05, ^b^*p* < 0.01, and ^c^*p* < 0.001.

## Discussion

This study examined the psychometric properties of the German version of the AIPC scale (Buck and Burster, 2017) in an online survey on COVID-19 contact tracing apps. The analysis included a confirmatory and exploratory factor analysis of the scale (factorial validity), tests of internal consistency (reliability), and correlations with barriers (convergent validity) and facilitators (discriminant validity) of contact tracing app use.

Overall, the study did not fully support the proposed three-factor structure of the scale. While the analyses mostly supported the factors titled *anxiety* and *personal attitude*, they did not replicate the factor titled *requirements*. Instead, the exploratory factor analysis pointed to two distinct factors of requirements concerning disclosure and control. Although the model suggested an overlap between aspects of anxiety and personal attitude, the latter two factors of requirements were independent. In their initial development of the scale, Buck and Burster (2017) cited Concerns for Information Privacy (Smith et al., 1996), Internet Users’ Information Privacy Concerns (Malhotra et al., 2004), and Mobile Users’ Information Privacy Concerns (Xu et al., 2012) as important groundwork. These models describe a variety of concerns regarding the collection, storage, use and secondary use of personal data as well as expectations and values of personal control, surveillance, and awareness of privacy practices. The AIPC synthesizes prior research and applies it to mobile app use, thus providing an important step in mHealth and health IT privacy development. The analysis resulted in the three factors of AIPC, namely anxiety, personal attitude, and requirements.

However, according to the exploratory factor analysis, a four-factor model was preferable, although this model requires further validation. In this model, factor 1 (information) was similar to personal attitude, which described the perceived importance of data protection and information privacy. Yet, it was also associated with anxiety (i.e., concerns about data use, processing, and storage) and factor 2 (data misuse) in this study, respectively. Possibly, the context of contact tracing apps might have introduced this association, because it connects health-related anxiety and privacy concerns (e.g., Gupta et al., 2021; Jenniskens et al., 2021; Kahnbach et al., 2021; Kolasa et al., 2021; Tomczyk et al., 2021; Grundy, 2022). The authors did not develop the AIPC as a health-specific measure of privacy concerns, thus factors like specific health concerns (e.g., Rosenstock, 1974; Rogers, 1975) were not included. In health behavior models, such as the protection motivation theory (Rogers, 1975) or the health belief model (Rosenstock, 1974), health-related concerns and risk perceptions have a longstanding tradition. According to these models, higher risk perception can lead to higher protection motivation and more protective behavior, for instance tracing app use (as a measure of infection prevention; Tomczyk et al., 2021). In the digital age, health concerns surpass physical or psychological health and also comprises digital health; hence, mobile health apps have to fulfill general privacy requirements but also health-related privacy requirements, particularly for vulnerable populations (e.g., Grundy, 2022). With COVID-19 being a genuine threat to the global population, health-related concerns might have conflated app-related privacy concerns and thus biased assessments of anxiety and personal attitudes. Nevertheless, the negative associations with adoption intentions and attitudes underline the importance of tailored health communication to address these aspects specifically when introducing mHealth apps and digitally supported infection prevention (Adjekum et al., 2018; Kahnbach et al., 2021).

Linking privacy concerns and health behavior modeling, it also seems important to discern requirements regarding disclosure and control (as observed in this study). Conceptually, these two aspects could differentially affect perceived control. In the theory of planned behavior, for instance, Ajzen describes two distinct facets of perceived behavioral control, self-efficacy and perceived controllability, that predict behavioral intentions (Ajzen, 1991, 2002). Self-efficacy refers to beliefs of individual performance ability and confidence (Bandura and Wessels, 1997), while perceived controllability refers to the beliefs of individual responsibility and opportunity. Thus, privacy practices, for instance, in disclosure policies, might affect perceived controllability, because they provide the setting for app use and data exchange. Control beliefs, however, are presumably linked to self-efficacy, because they refer to individual actions. In previous research, these constructs differentially affected health behaviors, such as help-seeking (Tomczyk et al., 2020). Hence, their association with privacy concerns warrants further attention.

Moreover, the observed overlap between items measuring anxiety and personal attitudes and the poor fit of the factor requirements receives further support from a study by Buck et al. (2018). In a series of experiments, they aimed to prime privacy concerns and thus incite changes in current perceptions of privacy concerns. In their study, the AIPC showed sufficient sensitivity to change, however, most manipulations affected anxiety and personal attitudes in a similar manner, and there were no significant effects on requirements. These experimental findings mirror the observations of this study, which suggest communalities of anxiety and attitudes, but not requirements. Applying a stronger contextualized focus to app information privacy concerns is therefore beneficial for future research.

Finally, the factors describing anxiety and personal attitudes showed good convergent and discriminant validity, which supports these dimensions of privacy concerns and corroborates previous findings (e.g., Smith et al., 2011; Xu et al., 2012; Dinev et al., 2015; Buck and Burster, 2017). Higher privacy concerns correlated positively with privacy victimhood, and negatively with attitudes and use intentions. And yet, the aspect of requirements (or disclosure and control) did not correlate with any of the variables. However, since privacy concerns were rather high, particularly requirements (*M* = 6.12, SD = 0.70, and range = 1–7), this limits possible statistical associations. Furthermore, explicitly stated privacy concerns as a barrier to tracing app use were not associated with AIPC scores, which challenges their validity. This conclusion is preliminary, because the proportion of participants who stated privacy concerns as a main barrier was rather small (=30/349).

### Strengths and limitations

The study investigated a cross-sectional German community sample, therefore it is not representative of the general population. It was also not possible to calculate test-retest reliability or sensitivity to change in this study. Cross-validation in new samples is recommended to inspect generalizability of the identified factor structure. Future studies could also extend psychometric examinations of the scale. The context of COVID-19 contact tracing apps provides an important yet specific scenario to study app information privacy concerns: As pointed out above, health concerns might play an important role in determining privacy concerns, which might not be the case in other use cases (e.g., online banking, e-commerce). This aspect is both a strength and a weakness of the study, because it provides a connection to mHealth research (e.g., Iwaya et al., 2020; Gupta et al., 2021; Hensher et al., 2021), yet it also leads to questions about the validity of these findings for different domains, and potential, health-related confounders (Hew et al., 2015). The study reported findings for different configurations of AIPC (three- and four-factor models). Nonetheless, future research could examine latent changes and measurement errors more closely in longitudinal models.

## Conclusion

The study aimed to test psychometric properties of the AIPC scale (Buck and Burster, 2017). While domains like anxiety and personal attitudes were confirmed in principle, the proposed factor structure was not supported. The analysis instead pointed to a substantial overlap between anxiety and personal attitudes, and a differentiation of requirements into external (disclosure policies) and internal (level of control) expectations of data handling. App information privacy concerns are an important issue in adoption and use of mHealth, as evidenced by negative associations with use intentions and attitudes toward COVID-19 contact tracing apps. And while general concerns and privacy-related attitudes seem to be well understood, motivational processes need further inquiry. Here, the connection between health behavior change, adoption and use of technology and privacy research, is promising.

## Data availability statement

The raw data supporting the conclusions of this article will be made available by the authors, without undue reservation.

## Ethics statement

The studies involving human participants were reviewed and approved by University Medicine Greifswald’s Ethics Committee. The patients/participants provided their written informed consent to participate in this study.

## Author contributions

ST conceived and designed the study, was responsible for data collection, and statistical analysis.

## Conflict of interest

The author declares that the research was conducted in the absence of any commercial or financial relationships that could be construed as a potential conflict of interest.

## Publisher’s note

All claims expressed in this article are solely those of the authors and do not necessarily represent those of their affiliated organizations, or those of the publisher, the editors and the reviewers. Any product that may be evaluated in this article, or claim that may be made by its manufacturer, is not guaranteed or endorsed by the publisher.
