# Supplementary material for: Appsolutely secure? Psychometric properties of the German version of an app information privacy concerns measure during COVID-19
Source: Front Psychol. 2022 Jul 22;13:899092. doi: 10.3389/fpsyg.2022.899092 (PMC9355691; doi:10.3389/fpsyg.2022.899092)
Supplement: Supplementary file 1 [file Data_Sheet_1.docx]

**Supplementary Tables**

**Supplementary Table S1**

Standardized factor loadings of a three-factor model of the App Information Privacy Concerns scale (N=349)

|  | Factor 1  („anxiety“) | Factor 2  („personal attitude“) | Factor 3  („requirements“) |
| --- | --- | --- | --- |
| Item 1 | 0.885 |  |  |
| Item 2 | 0.510 |  |  |
| Item 3 | 0.772 |  |  |
| Item 4 | 0.524 |  |  |
| Item 5 | 0.915 |  |  |
| Item 6 | 0.887 |  |  |
| Item 7 | 0.920 |  |  |
| Item 8 | 0.598 |  |  |
| Item 9 |  | 0.719 |  |
| Item 10 |  | 0.610 |  |
| Item 11 |  | 0.847 |  |
| Item 12 |  | 0.750 |  |
| Item 13 |  |  | 0.766 |
| Item 14 |  |  | 0.878 |
| Item 15 |  |  | 0.245 |
| Item 16 |  |  | 0.274 |
| Item 17 |  |  | 0.350 |

**Supplementary Table S2**

Standardized factor loadings of a four-factor model of the App Information Privacy Concerns scale (N=349)

|  | Factor 1  („information“) | Factor 2  („data misuse“) | Factor 3  („disclosure“) | Factor 4 („control“) |
| --- | --- | --- | --- | --- |
| Item 1 |  | 0.889 |  |  |
| Item 2 |  | 0.514 |  |  |
| Item 3 | 0.800 |  |  |  |
| Item 4 |  | 0.526 |  |  |
| Item 5 |  | 0.913 |  |  |
| Item 6 |  | 0.879 |  |  |
| Item 7 | 0.900 |  |  |  |
| Item 8 | 0.620 |  |  |  |
| Item 9 | 0.623 |  |  |  |
| Item 10 | 0.505 |  |  |  |
| Item 11 | 0.691 |  |  |  |
| Item 12 | 0.647 |  |  |  |
| Item 13 |  |  | 0.743 |  |
| Item 14 |  |  | 0.922 |  |
| Item 15 |  |  |  | 9.641 |
| Item 16 |  |  |  | 0.943 |
| Item 17 | 0.473 |  |  |  |
